# Metabolic Factors Associated with Endoscopic Atrophy, Intestinal Metaplasia, and Gastric Neoplasms in *Helicobacter pylori*-Positive Patients

**DOI:** 10.3390/clinpract14030062

**Published:** 2024-05-08

**Authors:** Junya Arai, Hiroaki Fujiwara, Tomonori Aoki, Ryota Niikura, Sozaburo Ihara, Nobumi Suzuki, Yoku Hayakawa, Masato Kasuga, Mitsuhiro Fujishiro

**Affiliations:** 1 Division of Gastroenterology, The Institute of Medical Science, Asahi Life Foundation, Tokyo 103-0002, Japan; hfujiwara29@gmail.com; 2 Department of Gastroenterology, Graduate School of Medicine, The University of Tokyo, Tokyo 113-8655, Japansozaburo.ihara@gmail.com (S.I.); nobu3szk@gmail.com (N.S.); hayakawayoku@gmail.com (Y.H.); mtfujish@gmail.com (M.F.); 3 Department of Endoscopy, Graduate School of Medicine, Tokyo Medical University, Tokyo 160-0023, Japan; rniikuratritonocn@gmail.com; 4 The Institute of Medical Science, Asahi Life Foundation, Tokyo 103-0002, Japan; m-kasuga@asahi-life.or.jp

**Keywords:** *Helicobacter pylori*, gastric atrophy, intestinal metaplasia, gastric neoplasms, fatty liver, diabetes mellitus

## Abstract

Background: Previous studies demonstrate an association between metabolic factors and *Helicobacter pylori*-related gastric cancer. However, the association of gastric atrophy or intestinal metaplasia (IM) with these factors remains unknown. Methods: Data on 1603 *Helicobacter pylori*-positive patients who underwent esophagogastroduodenoscopy between 2001 and 2021 were evaluated. The outcome measures were endoscopic atrophy, IM grade, and the incidence of endoscopically diagnosed and pathologically confirmed gastric neoplasms. Clinical factors associated with these findings were also determined. Results: Advanced age; successful *Helicobacter pylori* eradication; and comorbidities including diabetes mellitus (DM), hypertension, dyslipidemia, and fib4 index were significantly associated with endoscopic gastric atrophy grade. Male sex; advanced age; and comorbidities including DM, hypertension, dyslipidemia, hyperuricemia, fatty liver, aortic calcification, and fib4 index were also significantly associated with endoscopic IM grade, whereas advanced age, successful *Helicobacter pylori* eradication, DM, fatty liver, and fib4 index were significantly associated with the incidence of gastric neoplasms. Conclusion: Several metabolic disorders, including DM, hypertension, dyslipidemia, hyperuricemia, and fatty liver disease, are risk factors for advanced-grade gastric atrophy, intestinal metaplasia, and gastric neoplasms. Risk stratification according to these factors, particularly those with metabolic disorders, would affect EGD surveillance for *Helicobacter pylori*-positive patients.

## 1. Introduction

Gastric cancer (GC) is the third leading cause of cancer-related mortality worldwide [1]. The main carcinogen in gastric cancer is *Helicobacter pylori (H. pylori*), and its eradication prevents gastric cancer development [2,3]. The prevalence of *H. pylori* is influenced by the socioeconomic and health status of a country [4]. However, gastric cancer often occurs in *H. pylori*-eradicated patients, and several risk factors for *H. pylori*-related gastric cancer have been elucidated [3,4,5,6,7]. Currently, the association between gastric carcinogenesis and metabolic factors, mainly diabetes mellitus (DM) and fatty liver, is being focused on [3,5,6].

*H. pylori* infection could lead to gastric atrophy and intestinal metaplasia (IM). Moreover, the severities of both gastric atrophy and IM, estimated endoscopically or histopathologically, are associated with the incidence of gastric cancer [8,9,10,11]. Thus, these are considered precancerous lesions for gastric cancer. However, the association of gastric atrophy or IM with metabolic factors remains unknown. Given that gastric atrophy and IM are sometimes difficult to be improved by only *H. pylori* eradication, these precancerous conditions could be affected by additional contributing factors. We hypothesized that the grades of gastric atrophy and IM are also associated with metabolic factors, which can be the therapeutic target for GC prevention.

To address these issues, a single-center retrospective study was conducted to identify risk factors, particularly metabolic ones, for gastric atrophy, IM, and gastric neoplasms. This study aimed to elucidate the association between gastric atrophy/IM and metabolic factors.

## 2. Materials and Methods

### 2.1. Study Design, Setting, and Patients

**Figure 1:**
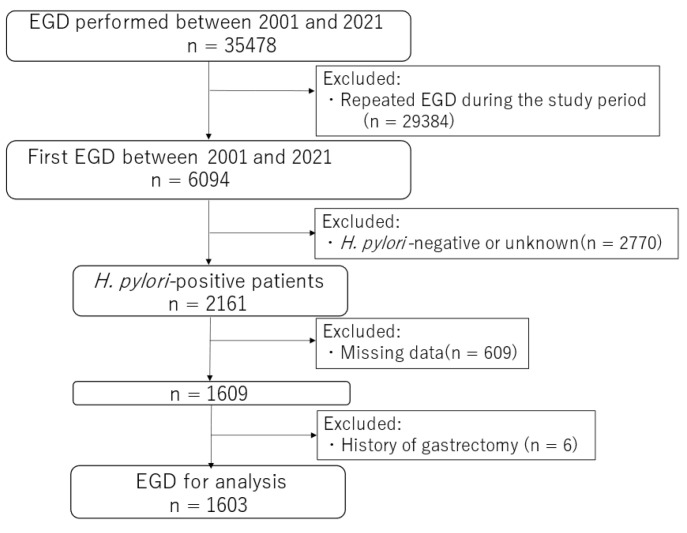
Flow chart of the patient selection process. Abbreviations: EGD, esophagogastroduodenoscopy.

All patient data were extracted from the endoscopic databases of the Institute of Medical Sciences, Asahi Life Foundation, Japan. Patients who underwent esophagogastroduodenoscopy (EGD), laboratory data analysis, and abdominal ultrasonography between 2001 and 2021 were evaluated. Patients who underwent repeat EGD during the study period and those with *H. pylori*-negative or unknown status, missing data, or a history of gastrectomy prior to the study period were excluded. *H. pylori* status was defined as the latest result of serological testing, a urea breath test, or a stool antigen test. Finally, 1603 patients were included in the analysis (Figure 1).

This retrospective study used the opt-out method. This study was approved by the Institutional Review Board of the Institute of Adult Diseases, Asahi Life Foundation (registration no. 15001, date of approval: 16 June 2023), and conformed to the provisions of the Declaration of Helsinki (as revised in Fortaleza, Brazil, October 2013).

### 2.2. Variables and Outcomes

The following clinical factors were evaluated as variables partly related to the risk of developing gastric neoplasms [3,5,6,10,11]: age, sex, waist circumference, successful *H. pylori* eradication, laboratory data at the first medical checkup reflecting the treatment naïve state [aspartate aminotransferase, alanine aminotransferase (ALT), alkaline phosphatase (ALP), ɤGTP, albumin, platelet, Creatinine (Cre), low-density lipoprotein cholesterol, high-density lipoprotein cholesterol (HDL), triglyceride, HbA1c, and uric acid (UA)], fatty liver scoring (Fib4 index, AAR, and APRI calculated in line with previous studies [12]), abdominal ultrasonography (fatty liver, gallbladder polyp, and aortic calcification), comorbidities [prescribed medications for DM, hypertension (HT), dyslipidemia (DL), and hyperuricemia (HUA)], and use of proton pump inhibitors.

The outcome measures were the grade of endoscopic atrophy, IM, and the incidence of gastric neoplasms. Endoscopic atrophy was estimated using the Kimura–Takemoto classification system and divided into four groups: non-atrophy, closed type 1/2 (C1-C2), closed type 3/open type 1 (C3-O1), and open type 2/3 (O2-O3) [13]. Endoscopic IM was also divided into four groups [grades 0, I (equivalent to atrophy C1-C2), II (equivalent to atrophy C3-O1), and III (equivalent to atrophy O2-O3)] according to the extended area of endoscopically detected IM, based on the Kyoto classification of endoscopic findings [14,15]. Gastric neoplasms included endoscopically diagnosed and pathologically confirmed adenomas and adenocarcinomas during the study period.

### 2.3. Statistical Analysis

Continuous variables were expressed as means with a 95% standard deviation, whereas categorical variables were expressed as numbers and frequencies (%). Continuous data were compared using analysis of variance. Categorical data between the groups were compared using the chi-square test or Fisher’s exact test, as appropriate. Prediction models for severe endoscopic atrophy (O2-O3), severe IM (grade III), and gastric neoplasms (adenoma and adenocarcinoma) were constructed with the associated factors using a logistic regression model. We did not opt to use the Harrell’s C statistic calculated with the Cox hazard model, as the data lacked information on the time course. Receiver operating characteristic (ROC) curves and estimated areas under the ROC curves (AUC) were plotted. The AUCs were compared between the prediction models with and without metabolic factors to estimate the impacts of these factors on gastric atrophy, IM, and gastric neoplasms. Each prediction model incorporated variables such as sex, age, and successful *H. pylori* eradication, which could potentially act as confounding factors for the outcomes. A *p* value of <0.05 was considered significant. All statistical analyses were performed using SAS software (ver. 9.4; SAS Institute, Cary, NC, USA).

## 3. Results

### 3.1. The Association among Endoscopic Gastric Atrophy, IM, and Gastric Neoplasms

Data from 1603 patients were analyzed. Advanced-grade gastric atrophy was significantly associated with advanced-grade IM and the incidence of gastric neoplasms. Advanced-grade IM is also associated with the incidence of gastric neoplasms. These results are shown in Table 1.

### 3.2. Factors Associated with Endoscopic Gastric Atrophy

Factors associated with endoscopic gastric atrophy are shown in Table 2. Among 1603 patients, 312, 640, and 608 were classified as C1-C2, C3-O1, and O2-O3, respectively. Age, successful *H. pylori* eradication, and laboratory data, including ALT, ALP, albumin, HbA1c, UA, Fib4 index, DM, HT, and DL, were significantly associated with the endoscopic gastric atrophy grade.

### 3.3. Factors Associated with Endoscopic Intestinal Metaplasia

The factors associated with endoscopic IM are presented in Table 3. Among 1603 patients, 453, 407, and 422 had IM grades I, II, and III, respectively. Male sex, age, and laboratory data, including PLT, HDL, HbA1c, UA, fatty liver, Fib4 index, DM, HT, DL, and HUA, were significantly associated with the grade of endoscopic IM.

### 3.4. Factors Associated with Gastric Neoplasms

Factors associated with gastric neoplasms are shown in Table 4. Among 1603 patients, 26 and 35 were diagnosed with adenoma and adenocarcinoma, respectively, during a mean follow-up period of 8.91 years. Age, successful *H. pylori* eradication, fatty liver, fib4 index, APRI, and DM were significantly associated with gastric neoplasms.

### 3.5. Prediction Model for Endoscopic Atrophy, Intestinal Metaplasia, and Gastric Neoplasms Using Several Variables

**Figure 2:**
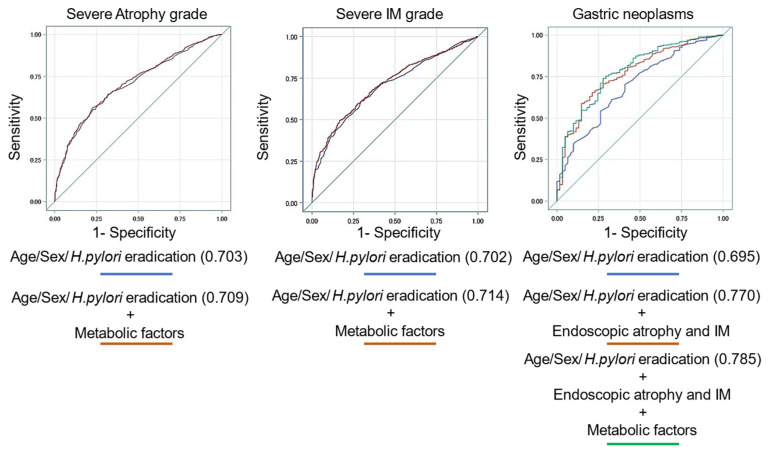
Prediction models for endoscopic atrophy, intestinal metaplasia, and neoplasms with several factors. The prediction model including sex, age, *H. pylori* eradication status, and metabolic fac-tors for endoscopic IM was significantly more accurate than that without metabolic factors but not for gastric atrophy. The prediction models using sex, age, *H. pylori* eradication status, endoscopic at-rophy/IM, and metabolic factors for gastric neoplasms were significantly more accurate than those without endoscopic features or metabolic factors.

**Figure 3:**
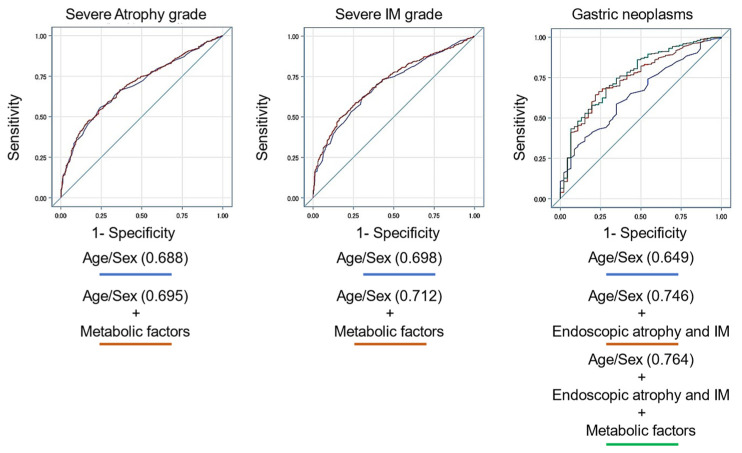
Prediction models for endoscopic atrophy, intestinal metaplasia, and neoplasms with several factors for *Helicobacter pylori*-eradicated patients. The prediction model including sex, age, and metabolic factors for endoscopic IM was significantly more accurate than that without metabolic factors but not for gastric atrophy. The prediction models using sex, age, endoscopic atrophy/IM, and metabolic factors for gastric neoplasms were significantly more accurate than those without endoscopic features or metabolic factors.

The results for endoscopic atrophy, intestinal metaplasia, and gastric neoplasms using several associated variables are shown in Table 5 and Figure 2. The prediction model including sex, age, *H. pylori* eradication status, and metabolic factors for endoscopic IM was significantly more accurate than that without metabolic factors (AUC: 0.714 vs. 0.702 [*p* = 0.029]) but not for gastric atrophy (AUC: 0.709 vs. 0.703 [*p* = 0.082]). The prediction models using sex, age, *H. pylori* eradication status, endoscopic atrophy/IM, and metabolic factors for gastric neoplasms were significantly more accurate than those without endoscopic features or metabolic factors (AUC: 0.785 vs. 0.770 vs. 0.695 [*p* = 0.005]). These results are similar to those for *H. pylori*-eradicated patients (Figure 3).

## 4. Discussion

This retrospective study found that age; successful *H. pylori* eradication; and laboratory data, including ALT, ALP, albumin, HbA1c, UA, Fib4 index, DM, HT, and DL, were significantly associated with the endoscopic gastric atrophy grade. Meanwhile, male sex; age; and laboratory data, including PLT, HDL, HbA1c, UA, fatty liver, Fib4 index, DM, HT, DL, and HUA, were significantly associated with the endoscopic IM grade. Age, successful *H. pylori* eradication, fatty liver, fib4 index, APRI, and DM were significantly associated with the incidence of gastric neoplasms. These factors improve the accuracy of the prediction models for endoscopic IM and gastric neoplasms.

DM or high serum HbA1c levels were associated with a higher risk of gastric atrophy, IM, and gastric neoplasms. Several studies demonstrate that DM and GC are associated [5,16]. The underlying mechanism relates to insulin resistance and hyperinsulinemia, which may stimulate the carcinogenic pathways of insulin. In both in vitro and in vivo studies, elevated glucose levels were observed to stimulate the growth and proliferation of GC cells and induce chemoresistance to 5-fluorouracil [17]. High glucose induces the expression of Nampt, Sirt1, p53, and P-gp and inhibits Top-IIα, which might lead to a poor prognosis of GC. This mechanism may affect the severity of gastric atrophy and IM. Further basic in vivo and in vitro experiments are required to understand the relationship between DM and alterations in the gastric mucosa.

In this study, fatty liver and fib4 index were independent risk factors for IM and gastric neoplasms. According to previous studies, fatty liver affects gastric carcinogenesis through enhanced insulin resistance; chronic inflammation with various signaling pathways, such as IL-6 and TNFα; adipocytokines; or alteration of gut microbiota [4,18]. These factors may also affect the severity of IM and the GC development. 

Several previous studies have shown that age, male sex, and *H. pylori* status are independent risk factors for IM and can be used as predictive models for IM [19,20]. However, metabolic factors, including DM, HT, DL, HUA, aortic calcification, fatty liver, and fib4 index, could improve the accuracy of predicting IM. Therefore, these factors should be considered during endoscopic surveillance according to the risk stratification of GC.

For the prediction model, IM grade and gastric neoplasms were more strongly associated with metabolic factors than with the atrophy grade. The exact reason for this is unknown, but these metabolic factors are independent risk factors for colorectal neoplasms [21,22]. Thus, metabolic factors might affect intestinal characteristics more strongly than gastric ones. However, further studies are required to elucidate this relationship.

For *H. pylori*-eradicated patients, metabolic factors also improve the accuracy of the prediction model for IM grade and gastric neoplasms. The GC development after *H. pylori* eradication is now being focused on [3,9,10], and metabolic factors might affect carcinogenesis by worsening the precancerous lesions even for successfully *H. pylori*-eradicated patients. Therefore, the treatment of these metabolic diseases might also be important to prevent GC development after *H. pylori* eradication. A prospective intervention study can be warranted to estimate whether the treatment of metabolic diseases could inhibit the progression of precancerous lesions.

In this study, IM staging was based on the range of endoscopically detected IM, similar to endoscopic atrophy. However, most studies usually estimated IM using the histopathological features of several biopsy specimens [8,9,10,11]. This endoscopic IM grade was also associated with both the atrophy grade and the incidence of neoplasms. Moreover, it is an accurate predictor of gastric tumors (AUC: 0.770) and is less invasive than endoscopic biopsies. Therefore, the endoscopic IM grade may easily help in risk stratification for GC.

Our study has several strengths. We evaluated the association between endoscopic features and various clinical factors, including waist circumference and laboratory data, which are generally difficult to obtain from endoscopic databases. However, our study has several limitations. First, this was a retrospective study, which can introduce selection bias and the presence of confounding variables. Second, the database had limited information regarding risk factors, encompassing lifestyle, genetic profiles, and medical data from other hospitals. Further prospective studies with a larger database are warranted. Third, the histopathological diagnoses of gastric atrophy and IM were not obtained. Especially, endoscopic IM might be underestimated, compared with histopathologically diagnosed IM. Further studies incorporating the pathological diagnosis of gastric atrophy and IM are essential for future research.

## 5. Conclusions

Several metabolic factors, including DM, HT, DL, HUA, fatty liver, and laboratory data, are associated with advanced gastric atrophy, intestinal metaplasia, and gastric neoplasms. Risk stratification according to these factors, particularly those with metabolic disorders, would affect EGD surveillance for *H. pylori*-positive patients.

## Figures and Tables

**Table 1:** Association among atrophy grade, IM grade, and gastric neoplasms.

Variables	No Atrophy (*n* = 43)	Atrophy C1-C2 (*n* = 312)	Atrophy C3-O1 (*n* = 640)	Atrophy O2-O3 (*n* = 608)	*p*
No IM (*n* = 321)	42 (97.67)	153 (49.04)	115 (17.97)	11 (1.81)	**<0.0001**
IM grade I (*n* = 453)	0 (0.00)	131 (41.99)	239 (37.34)	83 (13.65)	
IM grade II (*n* = 407)	1 (2.33)	24 (7.69)	190 (26.69)	192 (31.58)	
IM grade III (*n* = 422)	0 (0.00)	4 (1.28)	96 (15.00)	322 (52.96)	
No neoplasms (*n* = 1542)	42 (97.67)	310 (99.36)	628 (98.13)	562 (92.43)	**<0.0001**
Adenoma (*n* = 26)	0 (0.00)	1 (0.32)	5 (0.78)	20 (3.29)	
Adenocarcinoma (*n* = 32)	1 (2.33)	1 (0.32)	7 (1.09)	26 (4.28)	
Variables	No IM (*n* = 321)	IM grade I (*n* = 453)	IM grade II (*n* = 407)	IM grade III (*n* = 422)	*p*
No neoplasms (*n* = 1542)	319 (99.38)	444 (98.01)	391 (96.07)	388 (91.94)	**<0.0001**
Adenoma (*n* = 26)	0 (0.00)	4 (0.88)	5 (1.23)	17 (4.03)	
Adenocarcinoma (*n* = 32)	2 (0.62)	5 (1.10)	11 (2.70)	17 (4.03)	

Bold indicates *p* < 0.05. Abbreviations: IM, intestinal metaplasia.

**Table 2:** Factors associated with endoscopic gastric atrophy grade.

Variables	No Atrophy (*n* = 43)	Atrophy C1-C2 (*n* = 312)	Atrophy C3-O1 (*n* = 640)	Atrophy O2-O3 (*n* = 608)	*p*
Male	32 (74.42)	245 (78.53)	467 (72.97)	446 (73.36)	0.286
Age (years)	59.23 ± 14.74	59.89 ± 11.99	64.52 ± 10.35	70.54 ± 8.75	**<0.0001**
Waist	87.55 ± 9.80	90.05 ± 10.22	89.75 ± 9.71	89.88 ± 8.28	0.423
Successful *H. pylori* eradication	33 (76.74)	278 (89.10)	553 (86.41)	507 (83.39)	**0.036**
Laboratory data					
AST	21.86 ± 5.72	24.33 ± 12.86	23.93 ± 10.75	24.05 ± 13.91	0.673
ALT	22.30 ± 11.25	28.98 ± 21.05	27.10 ± 18.40	25.64 ± 17.88	**0.025**
ALP	202.93 ± 40.84	207.79 ± 70.15	218.01 ± 68.89	224.50 ± 79.27	**0.0051**
γGTP	38.74 ± 37.07	46.91 ± 49.03	49.03 ± 52.10	45.78 ± 59.84	0.534
Alb	4.39 ± 0.33	4.46 ± 0.27	4.44 ± 0.27	4.40 ± 0.26	**0.0058**
PLT	24.72 ± 6.23	24.29 ± 5.56	23.78 ± 5.03	23.51 ± 5.39	0.124
Cre	0.80 ± 0.18	0.79 ± 0.17	0.78 ± 0.17	0.78 ± 0.17	0.911
LDL	116.44 ± 31.47	120.35 ± 30.67	121.95 ± 29.43	117.98 ± 29.01	0.099
HDL	57.56 ± 15.68	56.24 ± 15.39	56.13 ± 15.64	54.26 ± 15.23	0.091
TG	140.79 ± 141.32	138.89 ± 113.63	146.55 ± 151.47	148.87 ± 150.94	0.786
HbA1c	6.53 ± 1.75	6.74 ± 1.92	6.86 ± 1.78	7.09 ± 1.89	**0.015**
UA	5.77 ± 1.42	5.62 ± 1.42	5.55 ± 1.37	5.33 ± 1.36	**0.0029**
AUS findings					
Fatty liver	29 (67.44)	232 (74.36)	480 (75.00)	453 (74.51)	0.750
Gallbladder polyp	15 (34.88)	120 (38.46)	226 (35.31)	224 (36.84)	0.805
Aortic calcification	2 (4.65)	24 (7.69)	48 (7.50)	63 (10.36)	0.205
Liver fibrosis score					
Fib4 index	1.24 ± 0.58	1.23 ± 0.57	1.36 ± 0.51	1.57 ± 0.77	**<0.0001**
AAR	1.11 ± 0.33	0.98 ± 0.36	1.02 ± 0.35	1.11 ± 1.14	0.053
APRI	0.29 ± 0.11	0.33 ± 0.19	0.33 ± 0.17	0.34 ± 0.26	0.345
Comorbidities					
Diabetes mellitus					**<0.0001**
Type 1	1 (2.33)	8 (2.56)	17 (2.66)	23 (3.78)	
Type2	13 (30.23)	124 (39.74)	315 (49.22)	369 (60.69)	
Hypertension	6 (13.95)	45 (14.42)	116 (18.13)	137 (22.53)	**0.017**
Dyslipidemia	3 (6.98)	34 (10.90)	81 (12.66)	104 (17.11)	**0.017**
Hyperuricemia	2 (4.65)	13 (4.17)	35 (5.47)	39 (6.41)	0.556
Medications					
Proton pump inhibitor	2 (4.65)	28 (8.97)	68 (10.63)	68 (11.18)	0.449

Bold indicates *p* < 0.05. Abbreviations: AST, aspartate aminotransferase; ALT, alanine aminotransferase; ALP, alkaline phosphatase; ALB, albumin; PLT, platelets; T-Bil, total bilirubin; Cre, creatinine; LDL, low-density lipoprotein cholesterol; HDL, high-density lipoprotein cholesterol; TG, triglycerides; UA, uric acid.

**Table 3:** Factors associated with endoscopic intestinal metaplasia grade.

Variables	No IM (*n* = 321)	IM grade I (*n* = 453)	IM grade II (*n* = 407)	IM grade III (*n* = 422)	*p*
Male	240 (74.77)	319 (70.42)	292 (71.74)	339 (80.33)	**0.0046**
Age (years)	59.64 ± 12.02	63.77 ± 11.05	67.34 ± 9.90	71.04 ± 8.22	**<0.0001**
Waist	89.03 ± 10.67	90.10 ± 9.42	89.96 ± 8.99	89.88 ± 8.31	0.424
Successful *H. pylori* eradication	273 (85.05)	385 (84.99)	347 (85.26)	366 (86.73)	0.877
Laboratory data					
AST	24.18 ± 15.43	24.32 ± 11.48	23.39 ± 11.41	24.09 ± 11.49	0.706
ALT	27.93 ± 18.35	27.02 ± 18.77	25.48 ± 18.70	26.94 ± 18.37	0.346
ALP	215.34 ± 69.24	216.01 ± 68.33	218.81 ± 75.95	221.68 ± 77.35	0.596
γGTP	48.29 ± 57.16	46.28 ± 43.90	45.29 ± 51.84	48.86 ± 63.72	0.764
Alb	4.44 ± 0.29	4.43 ± 0.26	4.41 ± 0.27	4.43 ± 0.27	0.402
PLT	24.30 ± 5.59	24.24 ± 5.45	23.44 ± 5.19	23.33 ± 5.00	**0.011**
Cre	0.79 ± 0.19	0.77 ± 0.18	0.79 ± 0.17	0.79 ± 0.15	0.219
LDL	120.81 ± 31.36	120.56 ± 29.71	122.04 ± 30.07	116.76 ± 27.43	0.061
HDL	58.49 ± 17.17	55.40 ± 15.46	55.14 ± 14.45	53.60 ± 14.71	**0.0003**
TG	135.58 ± 117.40	149.85 ± 159.93	143.32 ± 162.03	151.52 ± 125.95	0.432
HbA1c	6.85 ± 1.95	6.67 ± 1.75	6.89 ± 1.78	7.24 ± 1.93	**<0.0001**
UA	5.67 ± 1.35	5.58 ± 1.46	5.38 ± 1.38	5.34 ± 1.32	**0.0022**
AUS findings					
Fatty liver	219 (68.22)	338 (74.61)	314 (77.15)	323 (76.54)	**0.028**
Gallbladder polyp	108 (33.64)	169 (37.31)	144 (35.38)	164 (38.86)	0.477
Aortic calcification	22 (6.85)	27 (5.96)	37 (9.09)	51 (12.09)	**0.0075**
Liver fibrosis score					
Fib4 index	1.23 ± 0.60	1.35 ± 0.56	1.46 ± 0.57	1.57 ± 0.78	**<0.0001**
AAR	0.99 ± 0.35	1.04 ± 0.36	1.06 ± 0.33	1.10 ± 1.36	0.240
APRI	0.33 ± 0.23	0.33 ± 0.18	0.33 ± 0.19	0.35 ± 0.25	0.524
Comorbidities					
Diabetes mellitus					**<0.0001**
Type 1	9 (2.80)	7 (1.55)	15 (3.69)	18 (4.27)	
Type2	129 (40.19)	185 (43.93)	219 (53.81)	274 (64.93)	
Hypertension	37 (11.53)	76 (16.78)	86 (21.13)	105 (24.88)	**<0.0001**
Dyslipidemia	22 (6.85)	54 (11.92)	61 (14.89)	85 (20.14)	**<0.0001**
Hyperuricemia	12 (3.74)	19 (4.19)	22 (5.41)	36 (8.53)	**0.013**
Medications					
Proton pump inhibitor	29 (9.03)	50 (11.04)	40 (9.83)	47 (11.14)	0.746

Bold indicates *p* < 0.05. Abbreviations: AST, aspartate aminotransferase; ALT, alanine aminotransferase; ALP, alkaline phosphatase; ALB, albumin; PLT, platelets; T-Bil, total bilirubin; Cre, creatinine; LDL, low-density lipoprotein cholesterol; HDL, high-density lipoprotein cholesterol; TG, triglycerides; UA, uric acid.

**Table 4:** Factors associated with gastric neoplasms.

Variables	No Neoplasms (*n* = 1542)	Adenoma (*n* = 26)	Adenocarcinoma (*n* = 35)	*p*
Male	1138 (73.80)	22 (84.62)	30 (85.71)	0.133
Age (years)	65.52 ± 11.09	74.92 ± 8.81	69.86 ± 7.72	**<0.0001**
Waist	89.79 ± 9.31	88.85 ± 9.99	90.89 ± 8.71	0.687
Successful *H. pylori* eradication	1325 (85.93)	19 (73.08)	27 (77.14)	**0.041**
Laboratory data				
AST	23.90 ± 12.27	25.38 ± 13.15	27.09 ± 15.12	0.272
ALT	26.77 ± 18.73	25.50 ± 14.37	28.46 ± 17.92	0.816
ALP	217.85 ± 72.66	236.19 ± 84.30	214.89 ± 75.27	0.430
γGTP	46.82 ± 53.82	51.19 ± 63.87	56.54 ± 66.65	0.536
Alb	4.43 ± 0.27	4.38 ± 0.22	4.41 ± 0.23	0.654
PLT	23.84 ± 5.33	24.00 ± 4.80	21.75 ± 4.61	0.069
Cre	0.78 ± 0.17	0.80 ± 0.16	0.81 ± 0.13	0.681
LDL	119.95 ± 29.57	120.46 ± 26.01	121.14 ± 34.20	0.969
HDL	55.66 ± 15.53	51.58 ± 11.60	50.54 ± 13.94	0.066
TG	144.30 ± 144.65	179.08 ± 140.94	185.94 ± 125.87	0.119
HbA1c	6.91 ± 1.85	7.37 ± 2.38	7.02 ± 1.51	0.418
UA	5.48 ± 1.40	5.57 ± 0.93	5.46 ± 1.26	0.953
AUS findings				
Fatty liver	1141 (73.99)	23 (88.46)	30 (85.71)	**0.037**
Gallbladder polyp	562 (36.45)	10 (38.46)	13 (37.14)	0.975
Aortic calcification	134 (8.69)	3 (11.54)	0 (0.00)	0.165
Liver fibrosis score				
Fib4 index	1.40 ± 0.64	1.67 ± 0.62	1.77 ± 0.84	**0.0005**
AAR	1.05 ± 0.77	1.15 ± 0.51	1.07 ± 0.33	0.813
APRI	0.33 ± 0.21	0.34 ± 0.17	0.43 ± 0.36	**0.018**
Comorbidities				
Diabetes mellitus				**0.024**
Type 1	48 (3.11)	1 (3.85)	0 (0.00)	
Type 2	778 (50.45)	20 (76.92)	23 (65.71)	
Hypertension	291 (18.87)	5 (19.23)	8 (22.86)	0.837
Dyslipidemia	211 (13.68)	5 (19.23)	6 (17.14)	0.611
Hyperuricemia	84 (5.45)	3 (11.54)	2 (5.71)	0.404
Medications				
Proton pump inhibitor	159 (10.31)	3 (11.54)	4 (11.43)	0.958

Bold indicates *p* < 0.05. Abbreviations: AST, aspartate aminotransferase; ALT, alanine aminotransferase; ALP, alkaline phosphatase; ALB, albumin; PLT, platelets; T-Bil, total bilirubin; Cre, Creatinine; LDL, low-density lipoprotein cholesterol; HDL, high-density lipoprotein cholesterol; TG, triglycerides; UA, uric acid.

**Table 5:** Comparison of prediction model for endoscopic features with metabolic factors.

Prediction Model for Endoscopic Severe Atrophy (O2-3) Variables	AUC (95% CI)	*p*
Male, age, *H. pylori* eradication	0.703 (0.678−0.728)	0.082
Male, age, *H. pylori* eradication, DM, HT, DL, Fib4 index	0.709 (0.684−0.735)	
Prediction model for endoscopic severe intestinal metaplasia (grade III) Variables	AUC (95% CI)	*p*
Male, age, *H. pylori* eradication	0.702 (0.674−0.729)	**0.029**
Male, age, *H. pylori* eradication, DM, HT, DL, HUA, fatty liver, AC, Fib4 index	0.714 (0.688−0.741)	
Prediction model for endoscopic neoplasms (adenoma and adenocarcinoma) Variables	AUC (95% CI)	*p*
Male, age, *H. pylori* eradication	0.695 (0.629−0.760)	**0.005**
Male, age, *H. pylori* eradication, atrophy, IM	0.770 (0.713−0.828)	
Male, age, *H. pylori* eradication, atrophy, IM, DM, fatty liver, Fib4 index	0.785 (0.727−0.843)	

Bold indicates *p* < 0.05. Abbreviations: DM, diabetes mellitus; HT, hypertension; DL, dyslipidemia; HUA, hyperuricemia; AC, aortic calcification.

## Data Availability

Data supporting the findings of this study are available from the corresponding author upon reasonable request.
